# Quantification of parasite burden of *Trypanosoma cruzi* and identification of Discrete Typing Units (DTUs) in blood samples of Latin American immigrants residing in Barcelona, Spain

**DOI:** 10.1371/journal.pntd.0008311

**Published:** 2020-06-04

**Authors:** Maykon Tavares de Oliveira, Elena Sulleiro, Aroa Silgado Gimenez, Marta de Lana, Bianca Zingales, João Santana da Silva, J. Antônio Marin-Neto, Israel Molina

**Affiliations:** 1 Department of Infectious Diseases, Universitat Autònoma de Barcelona, Vall d’Hebron University Hospital. PROSICS, Barcelona. Spain; 2 Department of Internal Medicine, Cardiology Division, Medical School of Ribeirão Preto, University of São Paulo (FMRP-USP), Ribeirão Preto, SP, Brazil; 3 Department of Microbiology, Vall d’Hebron University Hospital. Universitat Autònoma de Barcelona_._ PROSICS Barcelona. Spain; 4 School of Pharmacy and Center for Research in Biological Sciences (NUPEB), Federal University of Ouro Preto (UFOP), Ouro Preto, MG, Brazil; 5 Department of Biochemistry, Institute of Chemistry, University of São Paulo (USP), São Paulo, SP, Brazil; 6 Department of Biochemistry and Immunology, Ribeirão Preto Medical School, University of São Paulo (FMRP-USP), Ribeirão Preto, SP, Brazil.; Universidade Federal de São Paulo, BRAZIL

## Abstract

**Background:**

*Trypanosoma cruzi* has a high genetic and biological diversity and has been subdivided into seven genetic lineages, named TcI-TcVI and TcBat. DTUs TcI-TcII-TcV and TcVI are agents of ChD in different regions of Latin America. Due to population movements, the disease is an emergent global public health problem. Thus, the aim of this study was to quantify the parasitic load and identify the presence of *T*. *cruzi* DTUs in 101 Latin American immigrants with chronic ChD, residing in Barcelona, Spain.

**Methodology / Principal findings:**

5ml of peripheral blood were collected in guanidine/EDTA from each patient for DNA extraction, quantification of the parasitic load and genotyping. A great variation of the parasitic load of the patients was verified: from 0.001 to 22.2 *T*. *cruzi* DNA (fg) / Blood DNA (ng). In patients from Bolivia the parasitic load was 3.76±4.43 *T*. *cruzi* DNA (fg) / Blood DNA (ng) (mean ± SD), in patients of other countries was 0.95±1.38 *T*. *cruzi* DNA (fg) / Blood DNA (ng). No statistically significant difference was observed in the parasitic load between patients with the indeterminate and cardiac forms of ChD (p = 0,57). Parasite genotyping was performed by multilocus conventional PCR. In patients from Bolivia there was a nearly equal prevalence of DTUs TcV (27/77), TcII/TcV/TcVI (26/77), and TcII/TcVI (22/77). TcVI was detected in only 2 samples (2/77). A higher prevalence of TcII/TcVI (19/24) was verified in patients of other countries, with low prevalence of TcII/TcV/TcVI (4/24) and TcV (1/24).

**Conclusions/Significance:**

In this study, low/medium parasitic load was found in all patients evaluated. Our data corroborate previous conclusions indicating that patients from the Bolivia, living in Spain, are predominantly infected by TcV, and TcVI DTUs. On the other hand, in Non-Bolivians patients TcII/TcVI predominated. Surprisingly, in our cohort of 101 patients no infection by TcI DTU was observed.

## Author summary

*Trypanosoma cruzi* is divided in seven distinct genetic groups (TcI-TcVI) and TcBat. They can be related to several biological parameters, the main being resistance to specific treatment. Due to the intense migration movements, ChD has become a serious public health problem in Europe. Thus, the work has the important function of identifying the genetic variability of *T*. *cruzi* circulating in the European continent, in addition to assessing the parasitic burden present in 101 chronic chagasic patients, residing in Barcelona, Spain. We show differences in the predominance between the infecting DTUs among Bolivian (TcV) and non-Bolivian patients (TcII/TcVI). This is the first study to describe the presence of TcVI genotype in Europe. Although the level of parasite burden is low/medium, it is higher in patients from Bolivia when compared with patients of other countries. The low parasitic burden is a limitation factor for studies aimed at evaluating by qPCR the effects of treating this disease with the drugs available to date, Benznidazole and Nifurtimox, and for clinical trials of new drugs. The information generated in this study should impact planning of more effective public health interventions to improve the health of chagasic patients, control vertical transmission and treatment of ChD.

## Introduction

Chagas disease (ChD) is caused by the hemoflagellate protozoan, *Trypanosoma cruzi* [1]. Approximately 60–70% of the chronic patients have no clinical symptoms (indeterminate form), whereas 30–40% either have or will develop cardiomyopathy, digestive megasyndromes or both [2]. According to the World Health Organization [3], 6–7 million people are chronically infected with *T*. *cruzi* worldwide, and more than 90 million individuals are at risk of infection. *T*. *cruzi* is genetically highly diverse and, at present, it has been subdivided into seven genetic lineages or discrete typing units (DTUs), named TcI to TcVI and TcBat [4,5]. *T*. *cruzi* DTUs have distinct, but not exclusive ecological and epidemiological associations [6]. With regard to ChD, DTU TcI is a major human infection agent in Amazonia, the Andean Region, Central America and Mexico, whereas DTUs TcII, TcV and TcVI are prevalent in patients in the Southern Cone region of South America [6–9].

In recent decades, the population movements from endemic to non-endemic countries have started to create notable changes in the epidemiology of ChD, as *T*. *cruzi* has spread worldwide [10,11]. The prevalence of ChD infection in Latin American immigrants living in Europe is estimated as 4.2%, with the highest prevalence among individuals from Bolivia (18.1%) and Paraguay (5.5%) [12].

Although direct vector transmission cannot occur in the European continent, infected blood transfusion, vertical transmission from mother to fetus and organ transplantation can provide parasite spreading in non-endemic countries [12]. Measures to control vertical transmission have been designed and implemented in some countries in Europe. However, these measures have not been effective [13].

Assessing the *T*. *cruzi* burden in immigrants from Latin America living in non-endemic countries has important implications for the implementation of medical care, monitoring of vertical transmission, introduction of additional controls for blood banks, training of personnel to diagnose and treat ChD, among others. In this direction, the present investigation aims at evaluating the parasitic load and the genotype of the infecting agent in immigrants from Latin America residing in Barcelona, Spain.

## Materials and methods

### Study population

This study included 101 ChD patients who were followed up by the clinical group of Infectious Diseases at Vall d'Hebron University Hospital, Barcelona, Spain, in the period 2015–2019. The patients had two positive serological tests for ChD, according to [3] and positive real-time PCR for *T*. *cruzi*. Patients were subjected to clinical evaluation consisting of anamnesis, ECG, resting transthoracic echocardiography, chest, esophageal and colon X-ray examination. The patients were classified into different clinical forms of chronic ChD, according to the [14]. Peripheral blood samples (5 mL) were collected and mixed with an equal volume of 6 M Guanidine Hydrochloride / 0.2 M ethylenediaminetetraacetic acid buffer (EDTA) solution, pH 8.0. The Guanidine-EDTA Blood lysates (GEB) were boiled for 15 minutes, incubated at room temperature for 24 h, and stored at 4˚C until use [15].

### Ethical clearance

The study was approved by the Human Research Ethics Committee of the Vall d'Hebron University Hospital. All patients provided written informed consent.

### DNA extraction

DNA was extracted from 200 μL of GEB samples and eluted with 55 μL of NucliSens easyMAG system (Biomerieux, France), according to the manufacturer's instructions.

### Parasitic load quantification by qPCR

The quantitative real-time PCR (qPCR) was performed according to a methodology previously proposed [16], using the multiplex *TaqMan* system targeting the 166 bp region of *T*. *cruzi* satellite DNA. The qPCR reactions were carried out at 25 μL final volume containing 5 μL DNA from each sample (20 ng/μL), 400 nM of the two primers and 100 nM of the TaqMan probe. The Quantitec Multiplex PCR kit (Qiagen, Manchester, United Kingdom) was used and the CFX Real-Time PCR detection system (Bio-Rad, Hercules, CA) used for amplification. The standard curve of the qPCR results was obtained using serial dilutions of 100 ng of DNA extracted from epimastigotes of the strain SO3 cl5 (DTU TcV), with a detection limit of 0.0001 fg, as proposed by [17] and modified by [18]. Positive, negative and reagent internal controls were used in all qPCR reactions.

### Genotyping of *Trypanosoma cruzi*

**Fig 1:**
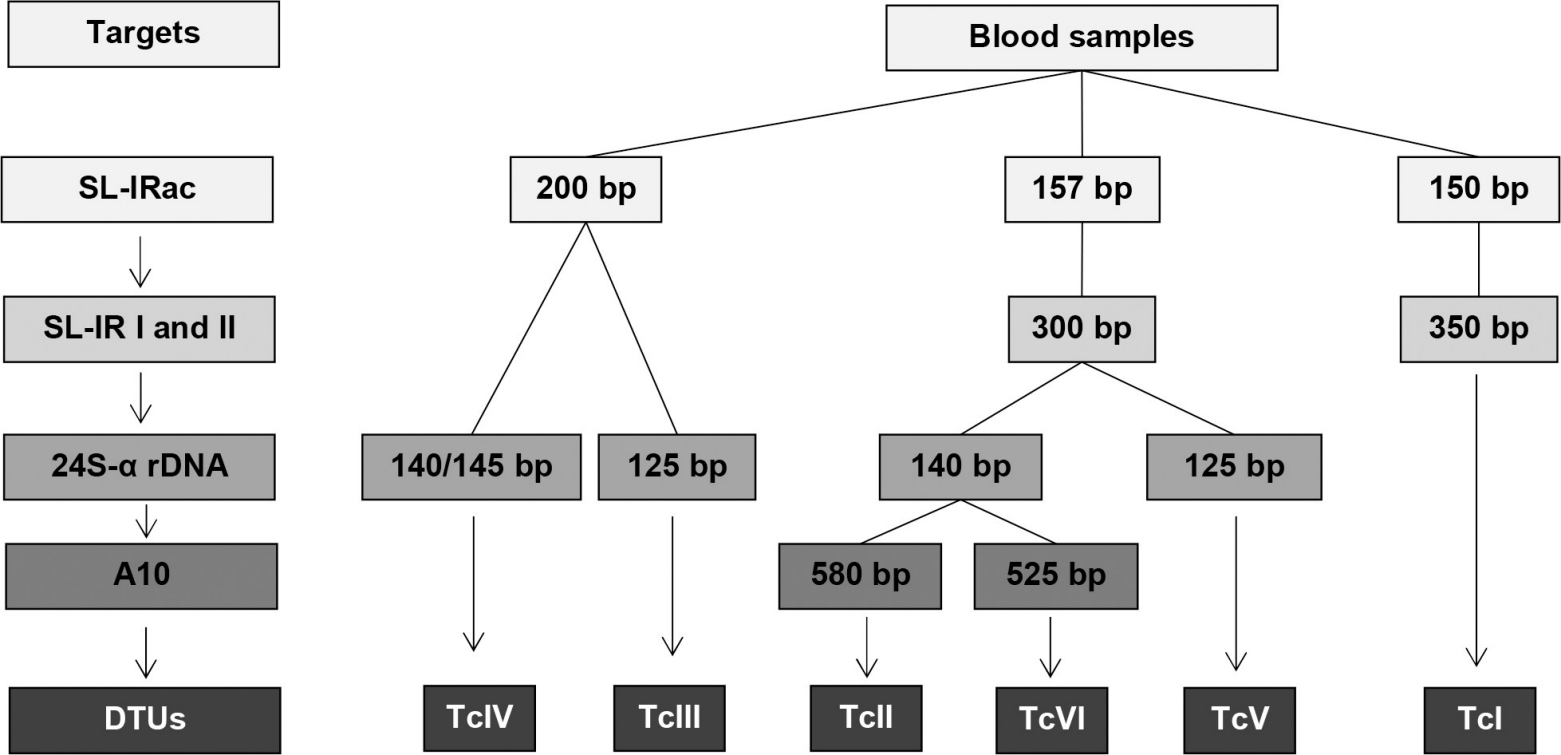
Multilocus conventional PCR for *Trypanosoma cruzi* genotyping in clinical samples [19]. The flowchart indicates the gene targets and the sizes of the PCR products expected for the DTUs.

Genotyping of *T*. *cruzi* in six DTUs (TcI-TcVI) was performed based on multilocus conventional PCR in association with Nested PCR, as described by [15] and modified by [19]. The subsequent identification of genotypes was based on the analysis of the set of profiles of the amplified PCR products presented for each gene target, using the following molecular markers: (i) the intergenic region of the Spliced Leader gene (SL-IRac) using the UTCC and TCac primers; (ii) the intergenic region of Spliced Leader (SL-IR) using TCC, TC1 and TC2 primers; (iii) the variable D7 domain of the 24Sα rRNA gene, with D75, D76 and D71 primers in semi-nested PCR; (iv) the A10 nuclear fragment in semi-nested PCR, with primers Pr1, P6 and Pr3. The PCR systems, gene targets and expected sizes of the amplified products are indicated in **Fig 1**. In all PCR reactions, DNA control samples from reference strains belonging to the six DTUs and Tcbat were used (Colombiana—TcI; Y—TcII; X109/2—TcIII; CanIII cl1—TcIV; Bug2148 cl1- TcV; CL Brener–TcVI and Tcbat 1994—Tcbat), as well as the negative controls and reagents. All amplification reactions were prepared in a final volume of 30 μL, using 12.5 μL of Mastermix Go Taq Green 2X (Promega, Madison, USA), 5 μL *T*. *cruzi* extracted DNA, and primers. The PCR cycling conditions were as described [15], using the Thermocycler (G-Storm, model GS 0001). The PCR products were separated by agarose gel electrophoresis (2% or 3% w/v), stained with Syber (Midori Green Advanced DNA Strain, Nippon Genetics Europe Gmbh) and viewed on Biorad photo documentation platform (Molecular Imager, Gel DOC XR, Imaging System). Molecular weight markers of 100 bp (Fast Gene Genetics, MWD100) were used to estimate the product size.

### Statistical analysis

All experiments were performed at least in two technical replicates. Categorical data were expressed as percentages, and continuous data as mean ± standard deviation (SD), or mean interval, according to the normality or nonparametric characteristic of the distribution. Student's t-test was used to analyze the significance of statistical differences. Results were deemed as statistically significant when p values were less than 0.05. Analysis was conducted using GraphPad Prism version 7.00 for Windows, GraphPad Software, La Jolla California USA, www.graphpad.com.

## Results

### Characteristics of the patients included in this study

This study included 101 patients with chronic ChD, not treated with Benzonidazole or Nifurtimox. All diagnosed by two positive serological tests and positive qPCR for *T*. *cruzi*, who were followed up at the Infectious Disease Clinic of the Vall d'Hebron University Hospital, Barcelona, in the period between 2015 and 2019. All patients reside in Barcelona and are immigrants from different countries of Latin America: Argentina (~ 8%), Bolivia (~ 77%), Brazil (~ 1%), Ecuador (~ 2%), Honduras (~ 1%), Paraguay (~ 4%), Uruguay (~ 5%) and Venezuela (~ 1%). Two patients were born in Spain, sons of Bolivian immigrants (**Table 1).** From the patients, 34 (33.7%) were male and 67 (66.3%) females (**Table 1).** The mean age was 48.2 years (24–80) **(Table 1).** The indeterminate form of ChD was diagnosed in 53 patients (52.5%) and 48 individuals (47.5%) presented the cardiac form. No patients with the digestive, nervous or mixed clinical forms of ChD were represented in our study population.

**Table 1:** General information of patients involved in the study and criteria for genotyping of *Trypanosoma cruzi*.

PATIENT INFORMATION						GENOTYPING CRITERION				
SAMPLE CODE	AGE (years)	GENDER	COUNTRY OF ORIGIN	CLINICAL FORM	PARASITE LOAD (*T*. *cruzi DNA (fg) / Blood DNA (ng)*)	TARGET GENES				DTU´S
						SLIR-ac	SL-IR I and II	24Sα rDNA	A10	
1	32	F	Bolivia	Cardiac	10.10	157bp	300bp	125bp	Neg	TcV
2	39	F	Bolivia	Indeterminate	2.49	157bp	300bp	Neg	Neg	TcII/TcV/TcVI
3	27	F	Bolivia	Indeterminate	6.74	157bp	300bp	125bp	Neg	TcV
4	43	M	Bolivia	Cardiac	6.75	157bp	300bp	125bp	Neg	TcV
5	43	M	Bolivia	Indeterminate	1.88	157bp	300bp	Neg	Neg	TcII/TcV/TcVI
6	65	F	Bolivia	Cardiac	1.52	157bp	300bp	125bp	Neg	TcV
7	49	F	Bolivia	Cardiac	6.21	157bp	300bp	Neg	Neg	TcII/TcV/TcVI
8	68	F	Bolivia	Cardiac	9.36	157bp	300bp	125bp	Neg	TcV
9	39	M	Bolivia	Indeterminate	1.41	157bp	300bp	125bp	Neg	TcV
10	60	F	Bolivia	Indeterminate	1.81	157bp	300bp	125bp	Neg	TcV
11	41	M	Bolivia	Indeterminate	4.57	157bp	300bp	125bp	Neg	TcV
12	39	M	Bolivia	Indeterminate	13.31	157bp	300bp	Neg	Neg	TcII/TcV/TcVI
13	43	M	Bolivia	Cardiac	1.29	157bp	300bp	Neg	Neg	TcII/TcV/TcVI
14	46	M	Bolivia	Indeterminate	3.21	157bp	300bp	125bp	Neg	TcV
15	43	F	Bolivia	Indeterminate	1.34	157bp	300bp	Neg	Neg	TcII/TcV/TcVI
16	38	F	Bolivia	Cardiac	3.98	157bp	300bp	125bp	Neg	TcV
17	56	F	Bolivia	Indeterminate	7.97	157bp	300bp	125bp	Neg	TcV
18	67	F	Bolivia	Indeterminate	1.00	157bp	300bp	125bp	Neg	TcV
19	59	F	Bolivia	Cardiac	4.22	157bp	300bp	140bp	525Pb	TcVI
20	35	M	Bolivia	Indeterminate	0.31	157bp	300bp	125bp	Neg	TcV
21	44	F	Bolivia	Indeterminate	0.98	157bp	300bp	125bp	Neg	TcV
22	56	F	Bolivia	Indeterminate	1.70	157bp	300bp	125bp	Neg	TcV
23	54	F	Bolivia	Cardiac	0.39	157bp	300bp	Neg	Neg	TcII/TcV/TcVI
24	28	M	Bolivia	Indeterminate	3.58	157bp	300bp	125bp	Neg	TcV
25	27	F	Bolivia	Indeterminate	8.97	157bp	300bp	125bp	Neg	TcV
26	26	M	Bolivia	Indeterminate	6.65	157bp	300bp	Neg	Neg	TcII/TcV/TcVI
27	39	F	Bolivia	Indeterminate	2.70	157bp	300bp	125bp	Neg	TcV
28	37	F	Bolivia	Indeterminate	5.78	157bp	300bp	125bp	Neg	TcV
29	56	F	Bolivia	Indeterminate	8.56	157bp	300bp	125bp	Neg	TcV
30	60	F	Bolivia	Cardiac	6.86	157bp	300bp	125bp	Neg	TcV
31	33	M	Bolivia	Indeterminate	3.10	157bp	300bp	140bp	525bp	TcVI
32	62	F	Bolivia	Cardiac	0.10	157bp	300bp	Neg	Neg	TcII/TcV/TcVI
33	44	F	Bolivia	Cardiac	4.81	157bp	300bp	Neg	Neg	TcII/TcV/TcVI
34	45	F	Bolivia	Cardiac	12.94	157bp	300bp	Neg	Neg	TcII/TcV/TcVI
35	47	M	Bolivia	Indeterminate	7.92	157bp	300bp	Neg	Neg	TcII/TcV/TcVI
36	53	F	Bolivia	Cardiac	1.93	157bp	300bp	125bp	Neg	TcV
37	71	F	Bolivia	Cardiac	2.24	157bp	300bp	Neg	Neg	TcII/TcV/TcVI
38	56	F	Bolivia	Indeterminate	5.30	157bp	300bp	125bp	Neg	TcV
39	64	F	Bolivia	Indeterminate	2.94	157bp	300bp	Neg	Neg	TcII/TcV/TcVI
40	64	M	Bolivia	Indeterminate	8.97	157bp	300bp	Neg	Neg	TcII/TcV/TcVI
41	44	M	Bolivia	Cardiac	5.05	157bp	300bp	125bp	Neg	TcV
42	80	F	Bolivia	Cardiac	20.87	157bp	300bp	Neg	Neg	TcII/TcV/TcVI
43	36	M	Bolivia	Indeterminate	1.87	157bp	300bp	Neg	Neg	TcII/TcV/TcVI
44	68	M	Bolivia	Indeterminate	1.85	157bp	300bp	Neg	Neg	TcII/TcV/TcVI
45	62	M	Bolivia	Cardiac	22.20	157bp	300bp	125bp	Neg	TcV
46	42	F	Bolivia	Indeterminate	10.70	157bp	300bp	Neg	Neg	TcII/TcV/TcVI
47	48	M	Bolivia	Indeterminate	6.14	157bp	300bp	Neg	Neg	TcII/TcV/TcVI
48	53	F	Bolivia	Cardiac	0.26	157bp	300bp	Neg	Neg	TcII/TcV/TcVI
49	43	F	Bolivia	Cardiac	0.98	157bp	300bp	125bp	Neg	TcV
50	45	F	Bolivia	Cardiac	0.79	157bp	300bp	Neg	Neg	TcII/TcV/TcVI
51	51	F	Bolivia	Indeterminate	1.02	157bp	300bp	Neg	Neg	TcII/TcV/TcVI
52	43	F	Bolivia	Cardiac	1.58	157bp	300bp	Neg	Neg	TcII/TcV/TcVI
53	50	F	Bolivia	Indeterminate	0.84	157bp	300bp	125bp	Neg	TcV
54	43	F	Bolivia	Cardiac	0.18	157bp	300bp	Neg	Neg	TcII/TcV/TcVI
55	39	F	Bolivia	Indeterminate	0.11	157bp	300bp	Neg	Neg	TcII/TcV/TcVI
56	49	M	Bolivia	Indeterminate	0.08	157bp	300bp	140bp	Neg	TcII/TcVI
57	37	F	Bolivia	Indeterminate	0.001	157bp	300bp	140bp	Neg	TcII/TcVI
58	49	M	Bolivia	Indeterminate	0.69	157bp	300bp	140bp	Neg	TcII/TcVI
59	70	F	Bolivia	Cardiac	0.11	157bp	300bp	140bp	Neg	TcII/TcVI
60	47	M	Bolivia	Indeterminate	5.89	157bp	300bp	140bp	Neg	TcII/TcVI
61	57	M	Bolivia	Indeterminate	0.67	157bp	300bp	140bp	Neg	TcII/TcVI
62	42	F	Bolivia	Indeterminate	7.40	157bp	300bp	140bp	Neg	TcII/TcVI
63	46	F	Bolivia	Cardiac	0.36	157bp	300bp	140bp	Neg	TcII/TcVI
64	42	F	Bolivia	Indeterminate	4.05	157bp	300bp	140bp	Neg	TcII/TcVI
65	70	F	Bolivia	Cardiac	2.24	157bp	300bp	140bp	Neg	TcII/TcVI
66	75	F	Bolivia	Cardiac	0.22	157bp	300bp	140bp	Neg	TcII/TcVI
67	39	F	Bolivia	Indeterminate	0.05	157bp	300bp	140bp	Neg	TcII/TcVI
68	45	F	Bolivia	Indeterminate	0.36	157bp	300bp	140bp	Neg	TcII/TcVI
69	69	F	Bolivia	Indeterminate	0.10	157bp	300bp	140bp	Neg	TcII/TcVI
70	33	F	Bolivia	Cardiac	1.23	157bp	300bp	140bp	Neg	TcII/TcVI
71	66	F	Bolivia	Cardiac	0.001	157bp	300bp	140bp	Neg	TcII/TcVI
72	37	F	Bolivia	Cardiac	0.13	157bp	300bp	140bp	Neg	TcII/TcVI
73	60	F	Bolivia	Cardiac	0.04	157bp	300bp	140bp	Neg	TcII/TcVI
74	35	F	Bolivia	Cardiac	0.47	157bp	300bp	140bp	Neg	TcII/TcVI
75	45	F	Bolivia	Cardiac	3.20	157bp	300bp	140bp	Neg	TcII/TcVI
76	52	M	Bolivia	Indeterminate	1.48	157bp	300bp	140bp	Neg	TcII/TcVI
77	67	F	Bolivia	Cardiac	0.43	157bp	300bp	140bp	Neg	TcII/TcVI
78	42	F	Argentina	Cardiac	1.46	157bp	300bp	Neg	Neg	TcII/TcV/TcVI
79	59	M	Uruguay	Indeterminate	4.66	157bp	300bp	Neg	Neg	TcII/TcV/TcVI
80	33	F	Argentina	Indeterminate	4.67	157bp	300bp	125bp	Neg	TcV
81	24	M	Paraguay	Indeterminate	0.10	157bp	300bp	Neg	Neg	TcII/TcV/TcVI
82	54	F	Brazil	Cardiac	0.17	157bp	300bp	Neg	Neg	TcII/TcV/TcVI
83	73	F	Paraguay	Cardiac	2.41	157bp	300bp	140bp	Neg	TcII/TcVI
84	63	M	Honduras	Indeterminate	1.25	157bp	300bp	140bp	Neg	TcII/TcVI
85	28	M	Uruguay	Cardiac	0.87	157bp	300bp	140bp	Neg	TcII/TcVI
86	47	M	Uruguay	Indeterminate	0.23	157bp	300bp	140bp	Neg	TcII/TcVI
87	42	F	Spain	Cardiac	0.78	157bp	300bp	140bp	Neg	TcII/TcVI
88	40	F	Paraguay	Cardiac	0.001	157bp	300bp	140bp	Neg	TcII/TcVI
89	69	M	Paraguay	Indeterminate	1.42	157bp	300bp	140bp	Neg	TcII/TcVI
90	47	M	Uruguay	Indeterminate	2.81	157bp	300bp	140bp	Neg	TcII/TcVI
91	42	F	Spain	Cardiac	0.001	157bp	300bp	140bp	Neg	TcII/TcVI
92	40	F	Argentina	Indeterminate	0.17	157bp	300bp	140bp	Neg	TcII/TcVI
93	39	F	Argentina	Cardiac	0.001	157bp	300bp	140bp	Neg	TcII/TcVI
94	32	F	Uruguay	Indeterminate	0.004	157bp	300bp	140bp	Neg	TcII/TcVI
95	43	M	Ecuador	Cardiac	0.48	157bp	300bp	140bp	Neg	TcII/TcVI
96	46	M	Argentina	Indeterminate	0.47	157bp	300bp	140bp	Neg	TcII/TcVI
97	43	F	Argentina	Cardiac	0.001	157bp	300bp	140bp	Neg	TcII/TcVI
98	42	M	Ecuador	Cardiac	0.74	157bp	300bp	140bp	Neg	TcII/TcVI
99	35	F	Venezuela	Cardiac	0.001	157bp	300bp	140bp	Neg	TcII/TcVI
100	64	F	Argentina	Cardiac	0.001	157bp	300bp	140bp	Neg	TcII/TcVI
101	43	M	Argentina	Cardiac	0.08	157bp	300bp	140bp	Neg	TcII/TcVI

F = Female, M = Male; bp = Base Pair; Neg = Negative; fg = Femtogram; ng = Nanogram; TcI, TcII, TcV and TcVI = *T*. *cruzi* genetic group; DTU´s = Discrete Typing Units.

### Parasitic load

**Fig 2:**
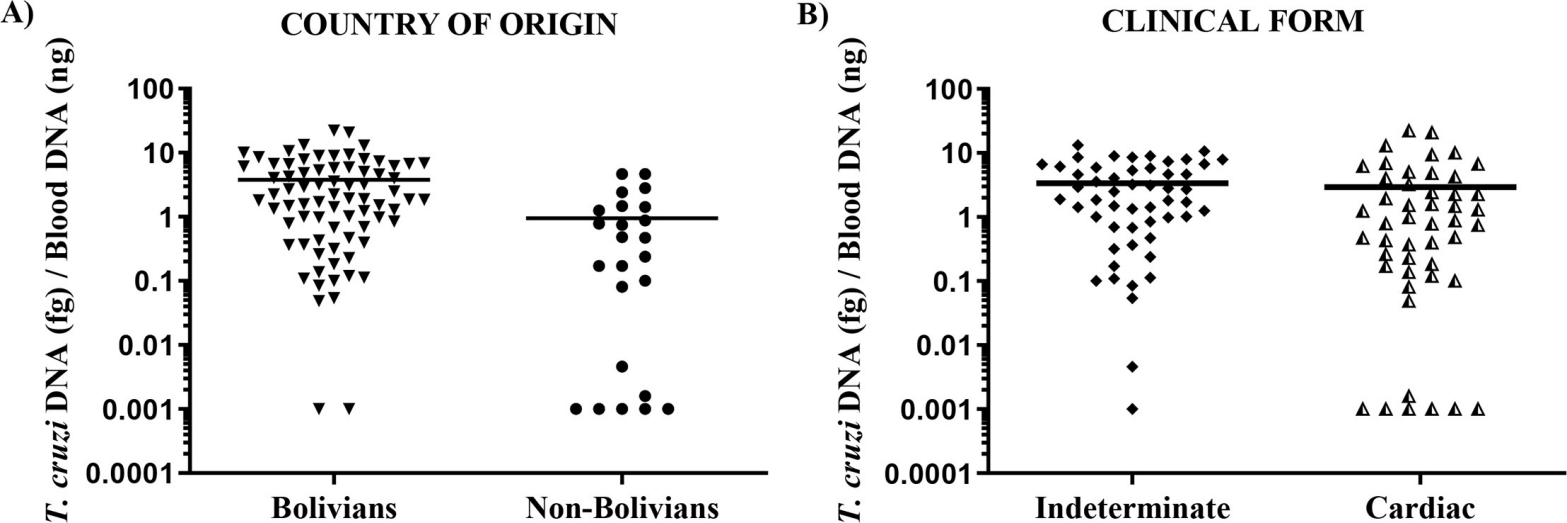
Parasitic load of Latin American immigrants with chronic ChD residing in Barcelona. Distribution according to (**A**) the country of origin; (**B)** the indeterminate and cardiac forms. The horizontal lines represent the mean values of the parasite load.

We observed a great variation of the parasitic load in the blood of the 101 patients: from 0.001 to 22.2 *T*. *cruzi* DNA (fg) / Blood DNA (ng). Regarding the country of origin, the mean ± SD of the parasitic load was 3.76 ± 4.43 *T*. *cruzi* DNA (fg) / Blood DNA (ng) in the Bolivian group and 0.95 ± 1.38 *T*. *cruzi* DNA (fg) / Blood DNA (ng) in the non-Bolivian group **(Fig 2A).** The data were statistically significant with a p value of 0.00029.

No statistically significant difference was observed in the parasitic load between patients with the indeterminate and cardiac forms of ChD **(Fig 2B).**

### *Trypanosoma cruzi* genotyping

**Fig 3:**
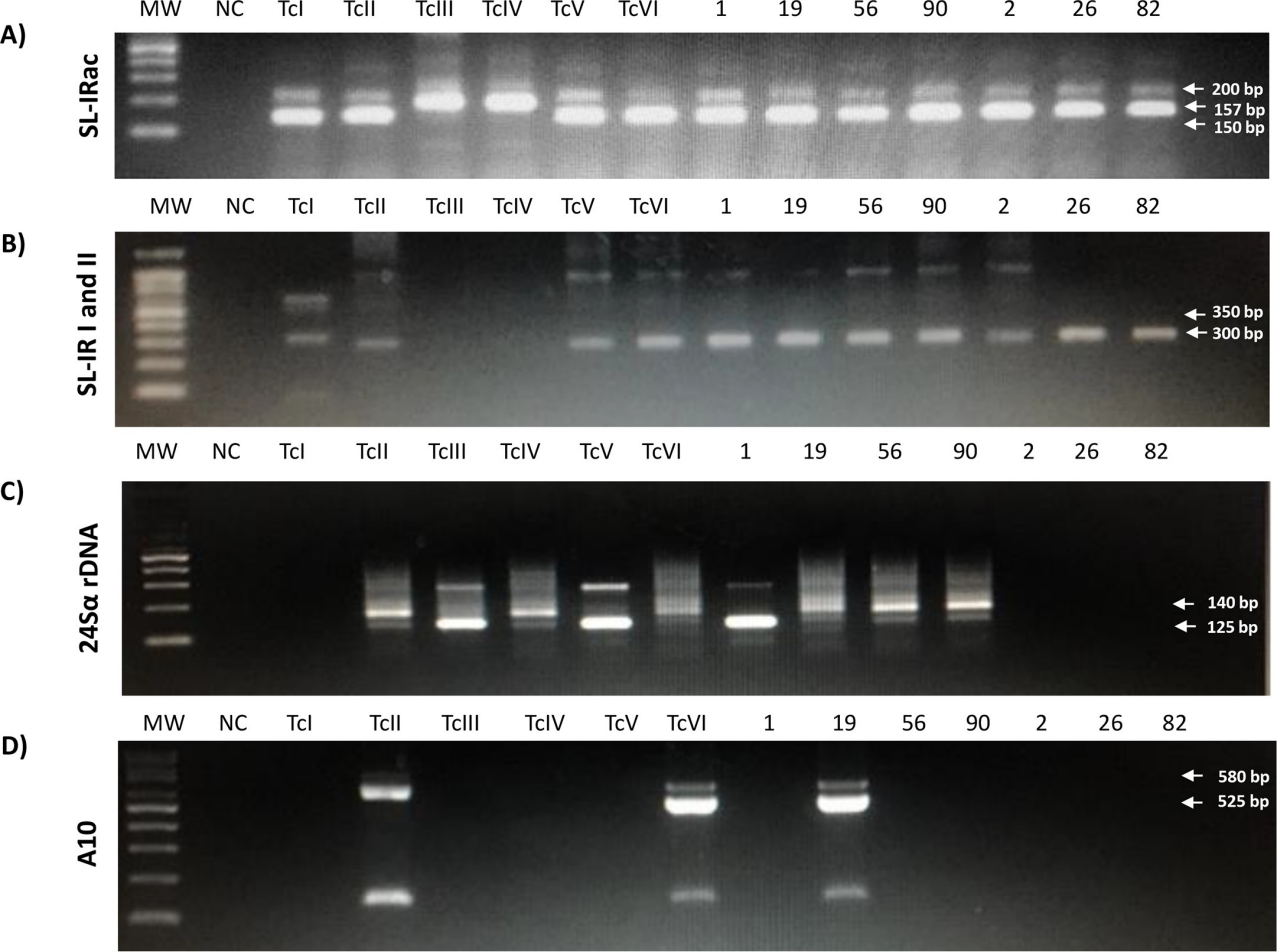
Representative gels of amplified gene products to define *Trypanosoma cruzi* DTUs. Genes: (A) the SL-IRac; (B) the SLIR I and II; (C) the 24Sα rDNA; (D) the A10. (MW—Molecular Weight marker; NC—Negative control; Positive controls, amplified products of reference strains: TcI, Colombiana; TcII, Y; TcIII, X109/2; TcIV, CANIII cl1; TcV, Bug2148 cl1; TcVI, CL Brener. The numbers indicate the code of the sample (see **Table 1**). Patient’s sample and infecting DTU: 1, TcV; 19, TcVI; 56, TcII/TcVI; 90, TcII/TcVI; 2, TcII/TcVI; 26, TcII/TcV/TcVI; 82, TcII/TcV/TcVI.

In all samples we applied the multilocus conventional PCR to perform the genotyping of the infecting DTUs. However, in samples which had a very low parasitic load we could not obtain amplified products of all the genes necessary for the molecular characterization of *T*. *cruzi* **(Fig 3; Tables 1 and 2).** In 27 DNA samples from Bolivian patients (27/77) the products confirmed DTU TcV infection, whereas in two Bolivian patients DTU TcVI was found. 22 Bolivian patients (22/77) had a genetic profile indicating infection by TcII/TcVI DTUs. In the remaining 26 patients from Bolivia (26/77) the amplified products suggested infection with TcII/TcV/TcVI DTUs.

**Table 2:** Genotyping of *Trypanosoma cruzi* from peripheral blood of chronic chagasic immigrant patients.

DTU´S	NUMBER OF PATIENTS	GEOGRAPHICAL ORIGIN
**TcV**	**28**	**Bolivia and Argentina**
**TcVI**	**2**	**Bolivia**
**TcII/TcVI**	**41**	**Argentina, Bolivia, Ecuador, Honduras, Paraguay, Spain, Uruguay and Venezuela**
**TcII/TcV/TcVI**	**30**	**Argentina, Bolivia, Brazil, Paraguay and Uruguay.**

The non-Bolivian patients (24/101) were infected by DTUs TcV (Argentina), TcII/TcVI (Argentina, Ecuador, Honduras, Paraguay, Spain and Venezuela) and TcII/TcV/TcVI (Argentina, Brazil, Uruguay and Paraguay) **(Tables 1 and 2).**

## Discussion

*T*. *cruzi* is composed of heterogeneous subpopulations that circulate in both domestic and wild cycles [20], and this diversity can be observed at the morphological [1,21], biological [22], antigenic [23] and at a genetic level [24,25]. Moreover, the parasite species are currently subdivided into seven distinct genetic groups (DTUs TcI–TcVI), and the Tcbat [4], with the additional fact that each DTU has its own characteristics [5]. In order to better understand the disease in each geographical region, it is important to study the molecular epidemiology of this parasite, which is naturally related to the main biological characteristics that have already been mentioned.

Currently immigration from Latin American countries to Europe has increased, especially in southern European countries such as Spain and Italy [26]. Since a considerable proportion of Latin American immigrants may be infected with *T*. *cruzi*, the epidemiology of ChD, originally endemic in Latin America, has changed considerably [10,27]. As a consequence, the number of reported cases of ChD with or without cardiac involvement has increased dramatically in recent years, especially in Spain, Italy and Switzerland [28,29].

In the present study we evaluated the parasitic load in the peripheral blood of 101 individuals serologically and real-time PCR positive for ChD, residing in Barcelona, Spain. Most of the patients were from Bolivia (77%). Patients from countries of the Southern Cone (Argentina, Brazil, Paraguay and Uruguay), northern South America (Ecuador and Venezuela) and Central America (Honduras) were also included. A wide variation of the parasitic load was observed among the patients and in most of them parasitemia was low / medium. Interestingly, the mean ± SD of the parasitic load of the Bolivian group (3.76 ± 4.43 *T*. *cruzi* DNA (fg) / Blood DNA (ng)) was higher than that of the group of patients from other countries (0.95 ± 1.38 *T*. *cruzi* DNA (fg) / Blood DNA (ng)).

In support to our conclusions, several studies employing quantitative real-time PCR (qPCR) have reported low / medium parasitic load values in chronic ChD patients of different countries of Latin America [30–33].

To investigate the impact of transfusion-acquired *T*. *cruzi* infection, [34] investigated blood donors who originated from Chagas-endemic areas and resided in the Mallorca Islands (Spain). Seropositivity for ChD was found in 23 (1.9%) of 1,201 donors and *T*. *cruzi* DNA with less than 1 parasite equivalent / mL was detected in the peripheral blood of 60.86% (14 of 23). Of the 14 patients in which circulating *T*. *cruzi* DNA was detected, 10 were from Bolivia, 3 from Argentina and 1 from Venezuela.

Higher parasitic load ranging from 1.43–11.14 parasite equivalents/mL (median 2.54) was reported in 65 chronic ChD patients from different regions of Brazil [15]. In a study similar to ours, the authors characterized the infectious DTU in 28 patients. They verified the prevalence of TcVI, TcII and mixed infection TcVI + TcII. When *T*. *cruzi* genotypes were compared with the parasite load, more elevated parasite loads were observed in patients infected by TcII (median of 7.56 par. Eq./mL) in comparison to patients infected by TcVI (median of 2.35 par. Eq./mL) [15].

In the present study we observed that patients from Bolivia (~77%) showed nearly equal prevalence of infections by TcV, TcII/TcVI and TcII/TcV/TcVI genotypes. In contrast, TcII/TcVI prevailed in patients from Argentina (~8%), Paraguay (~ 4%) and Uruguay (~ 5%). Of note, TcIII and TcIV were not identified in any patient, nor was TcI.

A previous study [35] also characterized the infecting DTU in peripheral blood samples of 10 migrants from Bolivia who attended hospitals in the Barcelona area. In agreement with our observations, in five samples TcV was identified; in three samples a TcII/V/VI profile was obtained and in the remaining two samples mixed infections TcV plus TcII/VI and TcV plus TcII was reported.

The DTUs infecting Latin American migrants attending a reference Clinic in Madrid was also defined [36]. As in our cohort, patients from Bolivia predominated (~90%). Overall, the most common DTU found was TcV (55.2%), followed by TcIV (16.2%), TcII (9.5%) and TcI (3.8%).

The scenario of the distribution of *T*. *cruzi* DTUs in ChD patients in countries of North, Central and South America has been outlined [5], based on data of [9] who surveyed articles in which approximately 6,400 DTUs were classified according to their geographical origin and hosts.

Our data regarding DTUs infecting migrants from Latin American countries residing in Barcelona follows the pattern of the geographic distribution of DTUs in the countries of origin. Two aspects stand out: To the best of our knowledge, this is the first study to describe the presence of TcVI genotype in the European continent in Bolivian patients with chronic ChD. TcI DTU was not found in any sample analyzed.

TcVI is highly related to the domestic cycle of ChD in some regions of the Southern Cone [6]. It is involved in human infections in the Chaco region in Northern Argentina; in Chile [37,38], and Brazil, more specifically in an outbreak of oral transmission in Santa Catarina state [39] and in endemic disease area in Minas Gerais state [40].

TcI DTU has a wide geographical distribution. TcI isolates are prevalent in patients from North America (Mexico and the United States); countries of Central America and northern South America (Colombia and Venezuela). Human TcI are abundant in Chile and the Brazilian Amazonia [5]. The fact that we did not find TcI in our cohort most probably is due to the low representativeness of individuals from Honduras and Venezuela or to the low abundance of this DTU in the sample.

We attempted to look for a possible association between the genotype of the parasite and the clinical presentation of ChD in the chronic phase. But, as discussed previously [5] we found none. We also found no correlation between the level of the parasite load and the infecting DTU.

Thus, knowing the parasite load and genetic variability of *T*. *cruzi* in chronic immigrant patients may be crucial to understanding the public health implications of ChD in European countries. Enhancing this understanding can allow for appropriate conception and planning of more effective public health interventions to improve the health of immigrants and control vertical transmission, which is a serious problem in European today.

### Conclusions

The data of this study corroborate previous reports indicating the prevalence of patients from Bolivia among the Latin American immigrants residing in Barcelona. We show differences in the infecting DTUs between Bolivian and non-Bolivian patients. This is the first study to describe the presence of TcVI genotype in European continent. Although the level of parasite burden is low / medium in the patients, it is higher in patients from Bolivia as compared with patients of other countries. The information generated in this study should impact planning of more effective public health interventions to improve the health of immigrants, control vertical transmission and treatment of ChD.
